# Logopenic aphasia or Alzheimer's disease: Different phases of the same disease?

**DOI:** 10.1590/S1980-57642014DN83000016

**Published:** 2014

**Authors:** Bárbara Costa Beber, Renata Kochhann, Bruna Matias da Silva, Marcia L. F. Chaves

**Affiliations:** 1 MSc, Dementia Clinic, Neurology Service, Hospital de Clínicas de Porto Alegre (HCPA), RS, Brazil.; 2 PhD, Post-graduate Program in Medicine: Medical Sciences, School of Medicine, Federal University of Rio Grande do Sul, Porto Alegre (UFRGS), RS, Brazil.; 3 CAPES Doctoral scholarship.; 4 Post-graduate Program in Psychology of the School of Psychology of the Pontifícia Universidade Católica do Rio Grande do Sul (PUCRS), RS, Brazil.; 5 CAPES Post-doctoral scholarship.; 6 Department of Internal Medicine, School of Medicine, UFRGS, RS, Brazil.

**Keywords:** logopenic aphasia, Alzheimer's disease, Primary Progressive Aphasia, diagnosis

## Abstract

The logopenic variant of Primary Progressive Aphasia, or logopenic aphasia, is a the most recently described variant of Primary Progressive Aphasia and also the least well defined. This variant can present clinical findings that are also common to Alzheimer's disease, given they both share the same cytopathologic findings. This article reports the clinical case of a patient for whom it proved difficult to define a clinical diagnosis, being split between the logopenic variant and Alzheimer's disease at different phases of the disease. Using this case as an example and drawing on the latest evidence from the literature on the logopenic variant, we postulate the hypothesis that this variant may present as an initial symptom of Alzheimer's disease in some atypical cases.

## Abstract (pt)

A variante logopênica da Afasia Progressiva Primária, ou afasia logopênica, é a variante mais recentemente descrita entre todas as variantes da Afasia Progressiva Primária e, também por isso, a menos definida. Essa variante pode apresentar achados clínicos em comum com a doença de Alzheimer pelo fato de compartilharem o mesmo achado citopatológico. Este artigo descreve o caso clínico de uma paciente na qual se evidenciou uma dificuldade em assumir o diagnóstico clínico que se dividia entre a variante logopênica e a doença de Alzheimer em determinadas fases da doença. Utilizando este caso como exemplo e as atuais evidências que a literatura apresenta sobre a variante logopênica, levantamos a hipótese de que essa variante pode apresentar-se como uma manifestação inicial da doença de Alzheimer em alguns casos menos típicos.

## INTRODUCTION

Primary Progressive Aphasia (PPA) is a term used to describe a group of neurodegenerative diseases that predominantly affect language.^1,2^ The term encompasses three different variants, each with a specific language profile: semantic, agrammatic/non-fluent and logopenic. The diagnosis of PPAs has long been restricted to the non-fluent and semantic variants, where logopenic aphasia has only recently been defined, based on the diagnostic criteria of Gorno-Tempini et al.^2^ The logopenic variant of PPA (lvPPA) is characterized by difficulties in single-word retrieval, repetition of sentences/phrases, presence of phonologic errors, left posterior perisylvian or parietal atrophy and typical association with the pathological finding of Alzheimer's disease (AD). Given that this variant has only recently been defined, descriptions of lvPPA and atypical cases remain relatively scarce, with fewer case studies and descriptions available compared to the other variants. Thus, the objective of this article is to report a clinical case for which it proved difficult to define a clinical diagnosis, being split between lvPPA and AD at different phases of the disease.

## CASE DESCRIPTION

We report the case of JCF, a 74-year-old female patient with 3 years of schooling, a native speaker of Brazilian Portuguese and housewife. The patient was referred to the Dementia Clinic of a University Teaching Hospital located in the south of Brazil in October, 2012. During the first visit, the patient was accompanied by her husband who provided all information owing to the her communication difficulties. The husband reported the main complaint as being a memory impairment which began in 2010. According to him, onset was abrupt and manifested with the forgetting names of people and objects, home address as well as her way of cooking. After a more in-depth review of the initial symptoms, the husband reported that the problems were predominantly saying the names of everyday objects properly and remembering how to write words, for instance, the patient would refer to a "glass" or pen" as "thing" because she was unable to recall the name of objects. However, the report was not consistent with impaired memory per se, particularly for the episodic type. Additionally, the husband reported a steady decline since onset of the "forgetful" condition, evidencing the progressive nature of symptoms. Yet despite this decline, he reported the patient continued to perform domestic chores, except for cooking, demonstrating some degree of independence in activities of daily living.

Before referral to the reference center, the patient had previously been assessed by a private neurosurgeon who gave no diagnosis but prescribed AAS 100mg, citalopram 10mg, and memantine 10mg. The patient had no medical history of previous systemic arterial hypertension (SAH), diabetes mellitus (DM), cerebral vascular accident (CVA), acute myocardial infarction (AMI), hospital admissions, smoking or alcohol dependence. For family history, it was reported that the patient's mother had died of cardiopathy (not specified), her father of pneumopathy (not specified), brother had died of cirrhosis and history of alcohol abuse.

At the Dementia Clinic, the patient was submitted to clinical, neurological and neuropsychological assessment. Until a diagnosis was established, memantine was withdrawn.

The neurological exam was unremarkable. The neuropsychological assessment was performed using the following tests with normative reference values for the Brazilian population: Mini-Mental State Exam (MMSE);^3^ Clinical Dementia Rating (CDR);^4^ Activities of Daily Living Questionnaire (ADLQ);^5^ Geriatric Depression Scale (GDS);^6^ The Consortium to Establish a Registry for Alzheimer's disease (CERAD)^7^ (for the sub-test with words list, the list was read out to patient who was not asked to read this as an alternative mode of the test); Hachinski Ischemic Score;^8^ Boston Naming Test (BNT);^7^ Digit Span Subtest (backward and forward) from the WAIS III;^9^ Clock Drawing Test (CDT);^10^ Montreal-Toulouse Language Assessment Battery (MTL),^11^ Phonemic Verbal Fluency (FAS)^12^ and Semantic Verbal Fluency (animals).^13^ The patient was unable to perform some of the tests owing to the difficulties exhibited (GDS and subtests Words list - recall and Words list - recognition from the CERAD).

The results of the tests applied are shown in Table 1, together with the expected scores based on normative reference values for the Brazilian population.

**Table 1:** Scores from cognitive assessment.

Tests performed		Patient score	Scoring range	Cut-off point for age and schooling
MMSE		4	0 to 30*	<22
CDR		2	0 to 3**	
ADL-Q		28	0 to 100***	
GDS		NPP	0 to 15 ****	
CERAD	Words list - fixation	0	0 to 30*	<13
	Words list - recall	NPP	0 to 10*	<3
	Words list - recognition	NPP	0 to 10*	<7
	Visuoconstructional praxis - copy	3	0 to 11*	<9
	Visuoconstructional praxis - recall	0	0 to 11*	<4
Hachinski Ischemic Score		1	0 to 12*****	
WAIS III - Digit Span	Forward	0	0 to 16*	< 2.74^#^
	Backward	0	0 to 14*	< 1.28^#^
BNT		4	0 to 12*	<12
CDT		0	0 to 5*	3
MTL	Automatic language - Form	6	0 to 6*	^##^
	Automatic language - Content	4	0 to 6*	^##^
	Repetition	11	0 to 33*	^##^
	Oral comprehension	14	0 to 19*	^##^
Verbal Fluency	Phonemic Verbal Fluency (FAS)	0	*	< 5.06^#^
	Semantic Verbal Fluency (animals)	1	*	< 7.65^#^

MMSE: Mini-Mental State Exam; CDR: Clinical Dementia Rating; ADL-Q: Activities of Daily Living Questionnaire; GDS: Geriatric Depression Scale; CERAD: The Consortium to Establish a Registry for Alzheimer’s Disease; BNT: Boston Naming Test; CDT: Clock Drawing Test; MTL: Montreal-Toulouse Language Assessment Battery; NPP: not possible to perform.

* Higher scores indicate better performance

** 0 (no dementia), 0.5 (questionable diagnosis), 1 (mild dementia), 2 (moderate dementia), 3 (severe dementia).

*** 0-33% = none to mild impairment, 34–66% = moderate impairment, 67+ % = severe impairment .

**** ≤5 = no depression, 6 to 10 = mild to moderate depression, >10 = severe depression.

***** 4-12: vascular dementia, 0-2: Alzheimer’s Disease, score 3: doubtful cases.

# this value means 1.5 Standard Deviations (SD) from the normal values for age and schooling.

## This value means 2.0 SDs from the normal values for age and schooling. A value of 2.0 SD was chosen because the normal values for schooling begin at 5 years and the patient had 3 years of schooling. The normative values were kindly provided by the authors of the battery, the publication of which is forthcoming.

In addition, an informal assessment of spontaneous speech was performed during the medical and cognitive assessment. No motor deficits or impairments in planning of speech motor acts, such as dysarthria or verbal apraxia were found. The patient also reported no swallowing complaints. Pauses during speech, word-finding difficulties and utterance of short sentences, as well as an absence of agrammatism were also observed. Comprehension difficulties were observed in situations involving complex speech but not when simple sentences and single words were used.

**Figure 1:**
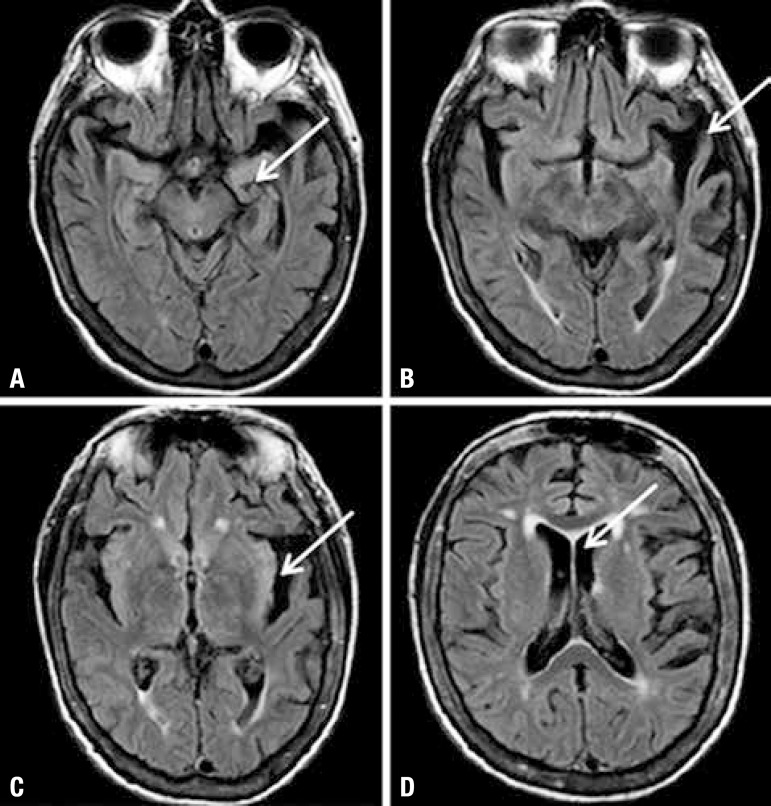
Axial FLAIR brain MRI Image reveals bilateral hippocampal reduction, predominantly to the left [A]; temporal lobe atrophy and extensive enlargement of Sylvian fissure, predominantly to the left [B and C]; Enlargement of CSF space [D].

**Figure 2:**
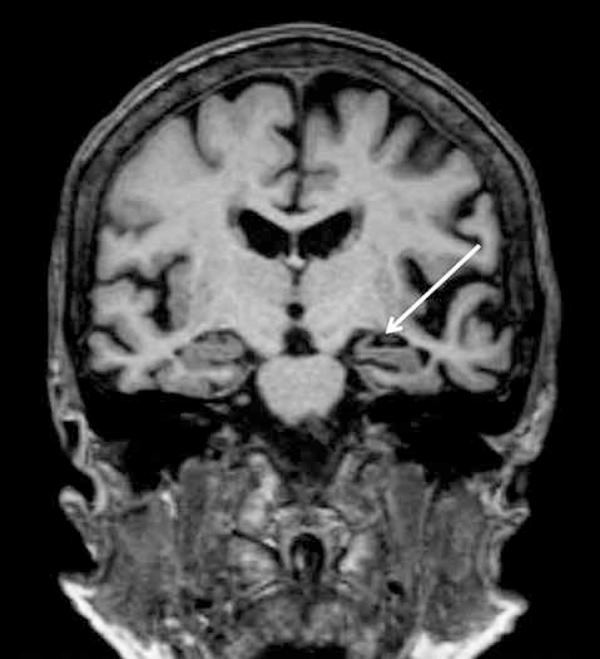
Coronal FLAIR brain MRI image. Image reveals bilateral hippocampal reduction predominantly to the left.

Laboratory exams and neuroimaging exams were ordered. The screening laboratory exams (full blood count, sera vitamin B^12^, VDRL, creatinine, TSH, etc.) revealed no abnormalities. In July 2012, a cranial computed tomography (CT) exam was performed revealing signs of left temporal lobe atrophy besides extensive left enlargement of the aqueduct of Sylvius. In April 2013, the patient was submitted to a brain MRI which disclosed bilateral hippocampal reduction and global enlargement of CSF spaces (Figures 1 and 2).

During the 4 visits by the patient over the 12 months of follow up, the management of medication was carried out with change in time of citalopram administration to the night period (as a result of excessive daytime drowsiness). Up to the last visit, anticholinesterasics had not been prescribed to the patient, who failed to return for the last visits scheduled.

## DISCUSSION

The main findings in this case study were the progressive aspect of the symptoms, predominantly language-related complaint, deficits on cognitive screening tests of the MMSE, CDR, verbal and non-verbal assessment of the CERAD, WAIS forward and backward digit span, the BNT, CDT, MTL (automatic language - content, repetition) and verbal fluency (phonemic worse than semantic) tests concomitant with relative sparing on the ADLQ and the oral comprehension test. On the informal assessment, key aspects that emerged included the absence of agrammatism and apraxia, and spared motor speech. The presence of cortical temporal atrophy and hippocampal reduction on MRI images were noteworthy.

According to reports by the patient's husband, the condition began with language symptoms but at the time of neurological assessment (2 years after first symptoms), the patient presented impaired memory and executive functions on cognitive assessment as well as language. The memory impairment displayed by the patient was evident on verbal and non-verbal assessment tasks from the CERAD battery and also on the memory domain of the CDR scale. However, it proved hard to distinguish to what extent the poor performance on verbal assessment was attributable to memory impairment or to aphasia.

Given the initial language-related symptoms, the diagnostic criteria for PPA^1^ and for lvPPA^2^ were reviewed and on which the patient fulfilled the necessary criteria, as shown in Chart 1.

**Chart 1:** Diagnostic criteria for PPA and lvPPA and criteria presented by patient.

**Diagnostic criteria for PPA^1^**	**Patient presented criterion?**
**Inclusion. Criteria 1-3 must be answered positively**	
1) Most prominent clinical feature is difficulty with language	Yes
2) These deficits are the principal cause of impaired daily living activities	Yes
3) Aphasia should be the most prominent deficit at symptom onset and for the initial phases of the disease	Yes
**Exclusion. Criteria 1-4 must be answered negatively for a PPA diagnosis**	
1) Pattern of deficits is better accounted for by other nondegenerative nervous system or medical disorders	No
2) Cognitive disturbance is better accounted for by a psychiatric diagnosis	No
3) Prominent initial episodic memory, visual memory, and visuoperceptual impairments	No
4) Prominent, initial behavioral disturbance	No
**Diagnostic criteria for lvPPA^2^**	**Patient presented criterion?**
**I) Clinical diagnosis. Both of the following core features must be present**	
1) Impaired single-word retrieval in spontaneous speech and naming	Yes
2) Impaired repetition of sentences and phrases	Yes
At least three of the following other features must be present	
1) Speech (phonologic) errors in spontaneous speech and naming	No
2) Spared single-word comprehension and object knowledge	Yes
3) Spared motor speech	Yes
4) Absence of frank agrammatism	Yes
**II) Imaging-supported lvPPA diagnosis. Both criteria must be present**	
1) Clinical diagnosis of lvPPA	Yes
2) Imaging must show at least one of the following results:	Yes, but not predominant. Presence of hippocampal atrophy SPECT and PET not performed.
a) Predominant left posterior perisylvian or parietal atrophy on MRI	
b) Predominant left posterior perisylvian or parietal hypoperfusion or hypometabolism on SPECT or PET	

lvPPA: logopenic variant of Primary Progressive Aphasia; MRI: magnetic resonance imaging; SPECT: Single-photon emission computed tomography; PET: positron emission tomography.

Although the patient met the diagnostic criteria for lvPPA, the diagnosis was not convincingly supported by the neuroimaging criteria which require predominant left posterior perisylvian atrophy on MRI, since the patient exhibited atrophy in this region together with hippocampal atrophy. Moreover, the patient presented a clinical feature of AD in the form of a deficit on the CERAD memory test (whose results may not have reflected true performance owing to the patient's aphasia picture) and age.

The hippocampal atrophy presented by the patient may be suggestive of typical AD. The first degenerative changes in AD occur in the medial temporal lobe including the hippocampus and entorhinal cortex,^14^ where hippocampal atrophy is described in studies using CT and MRI.^15-17^ However, many studies have also reported that hippocampal atrophy and atrophy of the entorhinal cortex can be present in other dementias, such as frontotemporal dementias and vascular dementiar.^18,19^ Hippocampal atrophy is the most well-established imaging biomarker for AD and has consequently been incorporated into new diagnostic criteria.

lvPPA on the other hand, is associated with greater left temporal lobe atrophy whilst the pattern of atrophy extends more posteriorly than that seen in the semantic variant, predominantly affecting the posterior perisylvian and temporoparietal regions (angular gyrus, posterior middle temporal gyrus, superior temporal gyrus and superior temporal sulcus).^20^ Unlike the majority of clinical symptoms associated with an asymmetric pattern of atrophy, logopenic aphasia is typically observed as a result of AD pathology.^2,21-23^

Typical AD and lvPPA tend to share the same pathological findings. The literature differentiates the latter as being an atypical presentation of the former, belonging to the spectrum of AD as an early form of presentation (Early Onset AD) with the language phenotype and atrophy predominantly in different brain regions, preferably assymetrical.^24^

Despite the clinical and pathological heterogeneity of the logopenic and agrammatic (non-semantic) variants of PPA, different clinical syndromes can be distinguished and correlated with a specific pattern of PIB-PET status. Phonological errors appear to be highly predictive of high amyloid load in PPA and may be a specific clinical marker for lvPPA. The study by Leyton et al.,^25^ besides the relationship with PiB-PET load, also showed that a different clinical profile characterized by anomia, impaired repetition of phrases, and more importantly, phonologic errors, can be identified within a broad category of lvPPA. The importance of phonologic errors as a predictor of AD pathology in PPA has been previously shown,^26^ but the criteria did not include them amongst the core diagnostic features.

On the other hand, although episodic memory impairment is the hallmark symptom of patients with amnestic type AD, this group may exhibit deficits in the semantic, syntactic and pragmatic components of language, but seldom present phonologic errors.^25^ Besides the linguistic aspects differentiating lvPPA from typical AD, the neuropsychological profiles of these patient groups differs, with a dissociation in performance of verbal and visual memory between the two conditions, where verbal memory is poorer in patients with lvPPA.^27^ These findings suggest that lvPPA has a different phenotype to AD.^25,27^

The case reported shows the difficulty determining a clinical diagnosis which was split between lvPPA and AD at certain phases of the disease. Based on the initial symptoms reported by the family member, language was clearly the first domain affected, lending support for a diagnosis of PPA. However, the patient had moderate dementia at the time of neurological assessment and her condition had evolved with presentation of not only language symptoms, but also non-verbal domains, while also exhibiting findings on neuroimaging exams suggestive of the clinical diagnoses of both lvPPA and AD. A cohort of patients with lvPPA showed that these patients presented rapid and generalized cognitive decline involving non-verbal domains, and the majority of cases met criteria for dementia within 12 months,^28^ similar to the pattern seen for the case reported in the present study.

Based on this case and on current evidence reported in the literature on lvPPA, we suggest that lvPPA may present as an initial symptom of AD in atypical cases. This clinical manifestation may occur due to the reliance that language mechanisms have on working and episodic memory, besides the neuroanatomic overlap that may take place between clinical presentations of typical AD and lvPPA given they share the same neuropathological findings. These aspects should be taken into account during the assessment and follow-up of atypical cases in order to better define the evolution, diagnosis and options for therapeutic management.
